# Perceptions of Mexican women regarding barriers in mental Heath Services in primary care

**DOI:** 10.1186/s12905-017-0423-x

**Published:** 2017-08-31

**Authors:** Jorge Galván, Nayelhi Saavedra, Feliciano Bartolo, Shoshana Berenzon

**Affiliations:** 1 Epidemiological and Psychosocial Research at the Ramón de la Fuente Muñiz National Institute of Psychiatry, Calzada México-Xochimilco 101. San Lorenzo Huipulco, Tlalpan, C.P.14370 Mexico City, Mexico; 2 Mental Health at the Federal District Public Health Services, Mexico City, Mexico

**Keywords:** Mental health care services, Barriers, Primary care, Women

## Abstract

**Background:**

The recent mental health care reforms in Mexico call for the regular evaluation of the services provided. This involves analyzing the opinions of those who utilize them on a daily basis, particularly women, since they are the main health service users. This study explores the barriers to mental health care perceived by a group of women attending primary care centers.

**Method:**

A qualitative methodological approach was chosen. The participants were purposively selected, using the snowball technique. Semi-structured interviews were analyzed using the thematic analysis.

**Results:**

Three sets of factors representing barriers to care were identified in the participants’ discourse. The first is linked to systemic barriers such as a lack of familiarity with the way the service operates, and irregularities in the consultations and appointment schedules that are not always geared to women’s needs. The second concerns the social stigma associated with emotional and/or mental disorders and their care while the third involves the characteristics of psychologists and their professional work.

**Conclusions:**

In order to overcome some of the barriers identified, users should be given information on the work of mental health professionals, which would help dispel certain misconceptions and sensitize them to the importance of this type of treatment in achieving overall health. There is also a need to make psychologists aware of the living conditions and socio-cultural context of the women who attend these health facilities.

**Electronic supplementary material:**

The online version of this article (doi:10.1186/s12905-017-0423-x) contains supplementary material, which is available to authorized users.

## Background

The Mexican health system comprises three subsystems. The first is the Mexican Social Security Institute, designed for salaried workers and funded by contributions from employees and employers. The second consists of a heterogeneous group of private services for those without health insurance. The third caters to the uninsured population which has no social security and the fewest resources [1, 2].

Primary health care centers (PH) providing services for the uninsured population are usually located in extremely marginalized areas. These centers are the first point of contact for a large sector of the population. They have poor infrastructure and insufficient human and material resources. These conditions are common in most of the health centers coordinated by the Mexico City Health Secretariat [3].

The population attending these health centers lives in precarious conditions in an environment of violence, overcrowding, addiction and unemployment. People in this environment are particularly prone to suffering from emotional and/or mental problems [4–6].

The health authorities have recently attempted to incorporate mental health services into PH in order to comply with the health policy promoted by the World Health Organization. This policy, a response to the increase in mental disorders in the world population, seeks to reduce the treatment gap [7–9].

In Mexico, the mental health care has yet to be fully incorporated into PH. The lack of financial resources, organization and coordination between the agencies responsible for mental health policies and services has meant that this process is still incomplete [10–15]. The problem is compounded by the fact that psychology and psychiatry services have never been a high priority at health centers.

This creates a series of obstacles to providing proper care. In the specialized literature, barriers arise as a result of the interaction between the characteristics of the health system, the provider and the individual and prevent the population from accessing health programs or services [16–18].

Most studies providing information on barriers to psychological and/or psychiatric care in the Mexican population were undertaken before mental health care was incorporated into PH institutions [4, 6, 19–23]. These studies identified barriers related to service characteristics such as accessibility, availability, and the gap between demand and service. Other barriers included users’ subjective evaluations of a particular disease and its treatment. This information has largely been provided by women, who are the main health service users [4, 6, 19–23].

This article seeks to identify the barriers perceived by a group of women in relation to mental health care at primary health care in Mexico City. It is important because it provides updated information on barriers to mental health care in light of the changes brought about by the recent policy of incorporating these services into PH. Besides, it reveals the opinions of a group of women vulnerable to emotional and mental suffering as a result of their precarious conditions, whose care needs are not usually taken into account by the health sector when strategies for improving the health system are designed.

## Methods

The information was obtained from an exploratory descriptive study derived from a broader study designed to analyze the strengths and weaknesses of mental health services at primary health care centers in Mexico City.

### Setting

The study was conducted at primary health care centers. They are classified as TI, TII and TIII [24, 25].

Sixteen centers were visited, five of which offered psychology service. There are two ways of obtaining access to this service; the first is through a General Practitioner (GP), who, suspecting that the patient may be suffering from a mental or emotional problem refers her to psychological services. The second is for the patient to directly make an appointment to see psychologist at the center, and ask social workers for to help with this procedure. When a center has a psychology service, a fixed number of appointments are assigned for consultation with a psychologist, as is the case with other services.

### Data collection

Contact with service users was achieved by two means: through nurses and social workers at the centers or by direct invitation while patients were waiting for consultation. Interviewees were purposively selected, using the snowball technique [24]. The inclusion criteria were as follows: a) being 15 years or older, b) regularly attending the health center c) agreeing to participate in the study.

We used a semi-structured format guide, with various thematic axes (see Additional file 1. For this paper we analyze the theme “Experience and use of mental health services”). The interviews were conducted by three of the authors of this manuscript in the waiting rooms of the Health Centers at times set by the respondents. The interviews lasted approximately 90 min.

#### Participants

Twenty women between the ages of 17–79 were interviewed. Most were housewives who had completed elementary school. They all declared that they lived in extremely marginalized neighborhoods and had a low socioeconomic status (Table 1).

**Table 1:** Socio-demographic data of the participants

Pseudonym	Age	Marital status	Occupation	Educational Level
U1	60 years	Widowed	Housewife	Elementary School
U2	39 years	Separated	Housewife	Vocational Degree
U3	17 years	Single	Student	Middle School
U4	60 years	Married	Housewife	Incomplete Elementary School
U5	49 years	Married	Housewife	Elementary School
U6	65 years	Married	Housewife	Elementary School
U7	70 years	Widowed	Housewife	Elementary School
U8	20 years	Living together	Housewife	High School
U9	79 years	Widowed	Housewife	Elementary School
U10	48 years	Married	Housewife	Middle School
U11	59 years	Married	Housewife	No Education
U12	73 years	Married	Housewife	Elementary School
U13	35 years	Married	Housewife	Middle School
U14	72 years	Widowed	Housewife	Middle School
U15	45 years	Married	Housewife	Middle School
U16	17 years	Living together	Housewife	Incomplete Middle School
U17	65 years	Married	Housewife	Elementary School
U18	27 years	Married	Housewife	Elementary School
U19	19 years	Single	Housewife	Incomplete Middle School
U20	55 years	Married	Housewife	Incomplete High School

The participants attended the health centers for a variety of reasons, such as taking their children to see a doctor, medication checks, monthly check-ups and ante-natal care. Although none of the participants cited mental illness as the reason that had brought them to see a doctor, the issue of mental health care emerged in the course of the interview.

### Data analysis

The audio-recorded interviews were transcribed. In order to analyze the information, thematic categories and subcategories were established to divide the narrations into information units to make it easier to understand the issues involved [26]. Analysis categories were established on the basis of the objectives outlined in the interview guides and each category was precisely defined to facilitate the encoding process. Two people encoded the interviews separately and discrepancies in the coding were resolved through the discussion and joint review of the original narrations.

In the results report, quotes taken from patients’ testimony were presented with a pseudonym in order to guarantee the confidentiality of the information.

### Ethical considerations

This study was approved by the Ethics Committee at the Ramón de la Fuente Muñiz National Institute of Psychiatry (IRB00006105). National [27] and international [28] ethical requirements were met. Due to the fact that this is regarded as a low-risk study, the participants’ consent was obtained verbally. Their permission was requested to audio record the interviews. Authorization was also requested to include the information provided as part of a general analysis to be presented at academic forums and/or in scientific publications.

## Results

The information presented below records some of the experiences of users, which, depending on their perception, become obstacles to accessing mental health services.

### Systemic barriers

#### Poor dissemination of the psychology service

The participants attributed their knowledge of the existence of the psychology services to the following: a) lack of dissemination of the service by personnel at the center through brochures or any other means and, b) lack of referrals by medical personnel to the mental health service. When one participant was asked about the department of psychology, she replied:

*“Well, it’s only now that you say so that I even know there’s a psychologist here…” [U5]*

#### Irregularity in consultations with the psychologist

One of the contributing factors to this situation is that psychologists are required attend training activities, meetings with authorities and events outside the center. These activities tend to crop up, meaning that it is impossible to cancel patients’ consultations in advance. Moreover, the number of psychologists is insufficient to meet demand, since there is usually one psychologist per center. The combination of these aspects creates irregularities in the service that often discourages patients from going to their consultations.

One patient noted: “*She gives us an appointment for more than a month later and sometimes I just don’t turn up” [U1]*

#### Inconvenient schedules for patients.

In order to meet patients’ care needs, some centers offer morning and evening service. According to the respondents, the lack of staff and/or the lack of safety in the area where the health center area is located makes it impossible to offer service in the evening.

Psychology service was offered exclusively in the mornings, which was inconvenient for many of the women who wanted to attend, since this is the time when most of them do their activities such as “going to the market”, “making lunch” or “picking up the kids”.

Moreover, we believe that the lack of importance given by the authorities to mental health care exacerbates the situation described above.

#### Extra expenditure caused by the lack of the service at the health center.

At health centers that do not have psychological care services when patients have an obvious emotional problem, they are referred to other institutions specializing in mental health. Participants who had been in this situation said that it entailed an extra financial burden, since they not only had to pay for transport to the institution to which they had been referred, but also for the consultation and their medication. These situations discourage patients from seeking care and/or lead to a lack of continuity in the care they receive.

When another woman, User 2, was unable to obtain mental health care at the health center, she decided to see a private psychologist, but had to give up treatment because she was unable to afford it.

*“Ah, because there are psychologists but private ones, some charge $50, $60 or $100 pesos for the consultation and if you don’t have enough money, well then it’s difficult and you prefer to just carry on as you are right now and not go. Because to be honest, I work and I earn very little, I mean I honestly can’t afford it, to take him to a private psychologist… I just´ can’t ” [U2]*

### Barriers related to social stigma

#### Negative perceptions surrounding psychological treatment

Consulting mental health specialists continues to be regarded negatively, either because of a lack of information, personal beliefs or prevailing attitudes within the participants’ socio-cultural context.

Some of the participants said that they had been worried about “what people would say” or that they had been ridiculed or criticized by family members, friends or neighbors after mentioning that they planned to see a psychologist. This type of experience was common and often served as a deterrent to seeking treatment. *“People misinterpret it, they start saying psychologists are for crazy people. Actually, the social worker here at the center, when I said they’d sent me to a psychiatrist, told me that the psychiatrists were for mad people and started laughing, and I was like… just like I am now, you see? I mean instead of supporting me, he just laughed.” [U3].*

#### The privacy of personal problems

The women expressed reluctance to discuss their problems with psychologists. For them, what happens in their families or to them is a private matter that should be protected, covered up and silenced ... They often use expressions such as, “Do not wash your dirty linen in public;” or “We shouldn’t even tell the priest what’s going on in our lives.”

The need to preserve privacy is related to the idea of avoiding embarrassment. When a person believes she is experiencing a situation that is outside socially accepted parameters such as having a husband who is cheating on her, having a child who is an addict or being a bad mother, she fears feeling humiliated or inferior to other people. For some women, the fear of being judged increases in the presence of people with authority or status, such as psychologists in their role as health professionals.

*“I don’t talk about my stuff, about what happens to me, and what goes on in my home stays in my home, that’s how I was raised, that’s why I don’t like talking to anyone about my problems and well, it can’t be helped, that’s the way it is.” [U10]*

### Barriers associated with health care providers

#### Mistrust of the treatment

The narrations analyzed showed that some of the women who attend the centers are unfamiliar with the mental health service. The scarce information they do have regarding the service is based mainly on the experiences of people in the community or their social imaginary that exists about psychologists.

Among the users at the centers we find that the most common ideas about what psychologists do are “guide,” “support” and “give advice and opinions” about people’s problems. These ideas tend to be confirmed by the media, which reinforces popular beliefs about psychologists’ work [29]. Activities such as guiding or giving advice, compared with those performed by doctors, nurses and social workers, may seem vague to patients, since they note that both family members and friends can offer advice and give their opinion.

That is, they do not think that, “Talking and giving advice” are activities requiring a professional, which is why they do not think it is useful to see a psychologist. “I don’t know if the psychologist worked (laughs), I went because of my baby, because I didn’t want to have it. But he just talked to me about the baby and said I had to have it and take care of it and, well, anyone could have told me that, couldn’t they? (laughs)” [U8].

#### Characteristics and attitudes of the psychologists.

As part of the social imaginary, there are a number of characteristics the Mexican population regards as desirable in a psychologist. The first is related to age; it is thought that the older a psychologist, the more experience he or she has. The second equates care with attitudes such as being kind, sensitive and understanding, all of which are usually associated with the female role.

Another has to do with the image of authority and is usually represented by a person who appears to have higher status than the patient (language, grooming, educational attainment). Most participants consider some of these attributes when they make the decision to start or continue psychological treatment. In the following quote, the participant refers to the second characteristic.

*“I didn’t mind the fact that he was a man, I didn’t have a problem with that. I didn’t like him because I felt like he was fussing, because I would come and talk to him and he’d say, “We’ve already talked about that” and maybe we had but I didn’t remember…” [U4].*

## Discussion

Three sets of factors representing barriers to care were identified in the respondents’ discourse. The first set is related to systemic barriers and as we mentioned in the introduction, a number of previous studies have reported similar obstacles [4, 6, 19–23].

We believe that the persistence of these barriers is due to the poor distribution of resources by the health sector. This in turn prevents the incorporation of mental health services into PH since fewer resources are assigned to this field.

Accordingly, too few psychologists and psychiatrists are hired and there is not enough room to give consultations or financing to publicize the services offered. Furthermore, given the small budget assigned to these institutions, resources are allocated to high-priority issues such as the treatment of diabetes, cancer and obesity and ante-natal care. Treating psychological disorders in PH remains a neglected area.

The second set of factors is related to the social stigma associated with emotional and/or mental disorders and their care. We believe that the low educational attainment of these women, in addition to the inaccurate information they share with other members of the community regarding mental illness and its treatment, helps reproduce the stigma towards those who use mental health care services.

As we noted in the introduction, most women live in overcrowded conditions, which leads to intense social interaction with their relatives and neighbors. This dynamic makes people protect the boundaries between what they allow others to know about them and their families and what they keep to themselves. They transfer this logic to the setting where the specialist takes the place of a stranger, who cannot be told everything because they fear he might disclose this information.

The third set of factors identified is related to psychologists and their work. We believe that the features mentioned by participants about what a psychologist should look like are drawn from the discourses and stereotypes that circulate in the media and are transferred to the population. One barrier arises when psychologists do not conform to type. This phenomenon has been observed in several countries in Europe, Latin America and the Caribbean [30, 31] .

One limitation of the study was the lack of a private space for conducting interviews with the participants. However, every effort was made to find the best conditions, such as relatively quiet areas in the waiting rooms away from the noise and presence of other patients.

Lastly, the authors were involved in the research process (protocol development, field work and information processing), which was a methodological advantage when it came to analyzing the data, since they had direct knowledge of the context of the interview and the participants, which enhanced the interpretation of the information.

## Conclusions

We believe that in order to overcome or at least mitigate some of the barriers identified; it would be advisable to assign more resources to services that cater to the general population. Primary health care users should be given information on the work of mental health professionals, which would help dispel misconceptions and sensitize them to the importance of this type of treatment in achieving overall health. Psychologists should also be made aware of the living conditions and socio-cultural context in which the participants live.

## Additional file

Additional file 1: Interview guide. (PDF 272 kb)

## Abbreviations

**PH**: primary care
**TI**: type one center
**TII**: type two center
**TIII**: type three center
