# Supplementary material for: Perceptions of Mexican women regarding barriers in mental Heath Services in primary care
Source: BMC Womens Health. 2017 Aug 31;17:70. doi: 10.1186/s12905-017-0423-x (PMC5580317; doi:10.1186/s12905-017-0423-x)
Supplement: Additional file 1: — Interview guide. (PDF 272 kb) [file 12905_2017_423_MOESM1_ESM.pdf]

Guía de entrevista a pacientes. (Interview Guide)

Buenos días/tardes:

Mi nombre es _____ y formo parte de un grupo de trabajo que está interesado en conocer algunas cuestiones relacionadas con las actividades que se realizan en las unidades del primer nivel de atención. Por esta razón, nos sería de gran utilidad conversar con usted acerca sobre sus experiencias y opinión sobre este centro.

La información que logremos recopilar será de gran utilidad para proponer acciones que podrían mejorar algunos de los servicios que se ofrecen en este tipo de centros. Todo lo que usted nos diga será confidencial, y será manejada de tal manera que nadie podrá reconocer quién dijo que cosa, por esta razón no es necesario que nos diga su nombre. Estas entrevistas generalmente son registradas en una grabadora, ya que de esta manera podremos tener una información más completa de lo que usted nos diga, ¿tendría usted algún inconveniente en que se grabará la entrevista?

Toda la información quedará resguarda del responsable del proyecto. La información obtenida se analizará de manera conjunta. Solicitamos su autorización para presentar este análisis general en foros académicos y/o publicaciones científicas. La entrevista tiene una duración aproximada de una hora y media.

En cualquier momento puede hacer preguntas sobre el procedimiento y en caso de desacuerdo podrá retirar su participación cuando usted lo desee y sin que esto afecte o tenga consecuencias en el servicio que recibe en el centro.

En caso de que tangan dudas o desee mayor información puede ponerse en contacto con la Dra. Shoshana Berenzon Gorn, responsable del proyecto, teléfono 41605168, correo electrónico berenz@imp.edu.mx.

¿Tiene alguna pregunta o comentario?

Le agradecemos de antemano su valiosa colaboración.

My name is _____ and I`m interested in knowing some issues related to the activities carried out in the health centers. For this reason, it would be very useful to know your experiences and opinion about this center.

The information that we collect will be very useful to propose actions that could improve some of the services offered in this type of centers. This information is confidential; you do not need to give us your name. You can ask questions about the procedure and in case of disagreement you can withdraw your participation when you want. This decision does not affect the service you receive at the center.

These interviews are usually recorded on a tape recorder, because this way we can have more complete information than you tell us, would you have any problem in recording the interview? The interview lasts approximately one and a half hours.

All the information will be kept by the project manager. The information provided as part of a general analysis. We request your authorization to present this general analysis in academic forums and / or scientific publications.

If you have any questions or would like more information you can contact Dr. Shoshana Berenzon Gorn, Project Manager, telephone 41605168, email berenz@imp.edu.mx.

Do you have any question or comment?

Thank you for your cooperation

EXPERIENCIA Y UTILIZACIÓN DE SERVICIOS DE SALUD (**EXPERIENCE AND UTILIZATION OF HEALTH SERVICES**)

Objetivo: Conocer los servicios utilizados por la población que acude a los centros de salud (Objective: To know the services used by the interviewers)

- Estrategias de autoatención (**Self-Attention Strategies)**
- Servicios alternativos (**Alternatives services**)
- Servicios formales (IMSS, ISSSTE, SS, Privado) **(Formal services)**
- Razones para utilizarlos (Para qué) **(Reasons to use formal or alternative services)**
- Razones por las que ya no lo utiliza o lo dejo de utilizar **(Reasons to not use formal or alternative services)**
- Opinión sobre cada uno de ellos **(Opinion of this services)**
- Comparación entre los servicios (ventajas, desventajas y función de los mismos) **Comparison between services)**
- Tipo de aseguramiento y características del mismo (cómo lo consiguió, trámites que realizó, etc.) (**Type of insurance**)
- Seguro Popular (conocimiento, expectativas, experiencias, etc.) **(Characteristics of Popular insurance)**
- Conocimiento de servicios de salud cercanos a su domicilio, trabajo o lugares que frecuenta (**Knowledge of health services near your home, work or places you frequent)**

1. RUTINA EN EL CENTRO **(PRIMARY CARE CENTER ACTIVITIES)**

Objetivo: Describir las actividades que realiza un usuario en el Centro de Salud (Objective: To describe the activities of the interviewees in the primary care centers)

- Descripción de un día de consulta en el Centro de Salud (desde que llego al centro hasta que salió del mismo) (**Description of a typical day in the center)**

- ¿Con cuáles trabajadores del Centro trató? **(Administrative and clinical staff consulted)**

2. EXPERIENCIAS EN EL CENTRO DE SALUD **(EXPERIENCES IN THE HEALTH CENTER)**

Objetivo: Conocer las percepciones y creencias que tiene el usuario sobre el centro de salud (Objective. To know the perceptions and beliefs about the health center)

- Conoce los servicios y actividades que ofrece este centro ¿por qué? (**Knowledge of the center's services and activities)**
- Razones por las que acude a este centro (razones personales/familiares) **(Reasons why you go to the health center)**
- Padecimientos por los que buscó/a consulta en este centro (razones personales/familiares) (**Diseases for which you seek help at the center)**
- Expectativas hacia la centro (qué esperaba y qué recibió**) (Expectations about center services)**
- **Experiencias sobre los servicios utilizados (Experiences about center services used)**
- Si pudiera cambiar algo de la forma en que se da el servicio ¿qué cambiaría? **(Change to improve services).**

3. EXPERIENCIAS DURANTE LA CONSULTA (**EXPERIENCES DURING THE CONSULTATION)**

Objetivo: Conocer las percepciones y creencias de los entrevistados sobre la consulta (Objective: To know the perceptions and beliefs of the interviewees about the consultation).

4. SALUD MENTAL EN LOS CENTROS DE SALUD (**MENTAL HEALTH CARE AT HEALTH CENTERS)**

Objetivo: Conocer las caracterìsticas de la atenciòn de la salud mental en los centros de salud. (Objective: To know the characteristic id mental health care in the health centers).

- Precepciones y creencias de la salud mental **(Perceptions and beliefs of mental health)**
- Información sobre los servicios de salud mental **(Information on mental health services)**
- Características de la atención a la salud mental **(Characteristics of mental health care)**
- Estigma y salud mental **(Stigma and mental health)**

5. SALUD MENTAL, REDES SOCIALES Y COMUNIDAD (MENTAL HEALTH, SOCIAL NETWORKS AND COMMUNITY)

Objetivo: Conocer la relación del contexto social y cultural con el cuidado de la salud mental. **(To know the relation of the social and cultural context with the mental health care.)**

6. DATOS SOCIODEMOGRÁFICOS (SOCIODEMIGRAPHIC CHARACTERISTICS)

Objetivo: Identificar las características sociales, culturales, educativas, etc., de la persona entrevistada**. (Objective: Identify the social, cultural and educational, characteristics of the interviewees.**
